# Going Cheap: Determinants of Bird Price in the Taiwanese Pet Market

**DOI:** 10.1371/journal.pone.0127482

**Published:** 2015-05-27

**Authors:** Shan Su, Phillip Cassey, Miquel Vall-llosera, Tim M. Blackburn

**Affiliations:** 1 Institute of Zoology, Zoological Society of London, Regent’s Park, London, United Kingdom; 2 Research Department of Genetics, Evolution and Environment, University College London, Gower Street, London, United Kingdom; 3 School of Biological Sciences, University of Adelaide, Adelaide, Australia; 4 Centre for Invasion Biology, Department of Botany and Zoology, Stellenbosch University, Stellenbosch, South Africa; INIBIOMA (Universidad Nacional del Comahue-CONICET), ARGENTINA

## Abstract

**Background:**

International wildlife trade is the largest emerging source of vertebrate invasive alien species. In order to prevent invasions, it is essential to understand the mechanics of trade and, in particular, which traded species are most likely to be released or escape into the wild. A species’ economic value is a key factor, because we expect cheaper species to be less assiduously secured against escaping, and more likely to be deliberately released. Here, we investigate determinants of the price of species in the Taiwanese bird trade. Taiwan is an international hub for bird trade, and several native species are threatened by alien bird species.

**Methodology:**

We investigated the relationship between the traded species sale price in Taiwan and the species availability for trade (the number of birds for sale, geographic range size and their origin, conservation and CITES status) and traits (body size, coloration, song attractiveness). We used phylogenetic generalized least squares models, with multi-model inference, to assess the variables that are best related to the price of birds in the Taiwanese pet trade.

**Principal Findings / Conclusions:**

We found that species available for sale in larger numbers, native to Taiwan, not globally endangered, and small-bodied are all relatively cheaper, as too are species lacking yellow coloration and without attractive songs. Our models of price revealed high levels of phylogenetic correlation, and hence that closely related species tended to be sold for similar prices. We suggest that, on the basis of price, native species are more likely to be deliberately or accidentally released than alien species. Nevertheless, our survey of bird shops recorded 160 species alien to Taiwan (7,631 individuals), several of which are for sale cheaply and in large numbers. Alien bird species in trade therefore present an ongoing, non-trivial invasion risk on the island.

## Introduction

Geographical barriers that have naturally set limits species distributions are increasingly being breached as species are moved around by international trade [1–4]. This is a cause for considerable concern because alien species can have substantial negative impacts on the recipient regions [5–7], including causing the extinction of native species [6,8] and the homogenization of ecological assemblages (with a concomitant decrease in species diversity) [9].

One of the major pathways of human-mediated movement is the trade in wild animals and plants, including the trade in cage birds [10]. Captivity places constraints on the invasion process [4,11], because traded species are not directly introduced after transport and not all species in trade are introduced. Nevertheless, the wildlife vertebrate trade is an increasing source of invasive species [12–14]. Alien species in trade have been shown to be correlated, in terms of identity [15] and abundance [16], with those species that subsequently establish alien populations. Indeed, the most important correlate of variation in the number of invasive alien species across countries is currently the volume of merchandise imports [17], suggesting that international trade is now the primary driver of species invasions (see also Colunga-Garcia *et al*. [18]). To characterise invasions effectively, it is therefore important to understand the mechanics of the wildlife trade, and in particular, which traded species are most likely to be released or escape into the wild.

The amount of care devoted to keeping a bird is likely to be associated with its value [19]. Therefore, we have assumed that sale price is one of the factors that will influence the likelihood that traded species are introduced into the wild. More valuable species are expected to receive better care and hence to be more assiduously secured against escaping, and less likely to be deliberately released by their owners. Here, we use the example of the cage bird trade in Taiwan to explore reasons for variation in the price of species for sale.

Taiwan presents an excellent example for studying features of the bird trade in Asian countries [20]. First, cultural practices relating to the bird trade, such as prayer release, singing competitions and ‘bird-walking’ (the avian equivalent of dog-walking, where birds are taken out in cages for fresh air), are very popular in Taiwan. Prayer animal release is a common religious activity in Asia [20]: it is estimated that more than 200 million animals are set free through this practice in Taiwan every year [21,22]. Such a high frequency of releases may be an important factor contributing to invasions by alien bird species [5,23–26]. Second, Taiwan is a major wildlife trade hub from where species are re-exported to other Asian countries, such as Malaysia and Singapore [20,27,28]. Third, Taiwan is separated from the nearest continent by a >100 km ocean strait, meaning that it is relatively straightforward to define native versus alien species. Finally, alien bird species have proved to be a conservation threat in Taiwan through actual and potential hybridization with native bird species [29–31].

We predicted that the price of traded birds in the Taiwanese pet market would largely be determined by a species’ availability, because supply costs would be lower for common species, and because people will pay higher prices for rarer species (the anthropogenic Allee effect [32]). We therefore hypothesised a negative relationship between price and the number of individuals of a species offered for sale. Species that are more widespread are normally more abundant [33,34], and more available for trade [24]. We hypothesised that species that have more extensive native distributions would be less expensive. Similarly, we hypothesised that native species and species coming from closer regions, species not considered as threatened on the IUCN Red List, or species not listed on CITES Appendices, would also be cheaper. However, species availability is not the only factor suggested to affect market value. An extensive body of literature has shown that particular species traits can affect human attitudes towards species, including willingness to pay. Animal body size is a trait that influences human preference [35], and larger-sized species are more valued [36]. Body size is negatively related to bird species richness [37,38] and abundance [34,39], both of which may affect the availability of large-bodied birds for sale. Large-bodied birds also tend to be longer-lived and reproduce more slowly, and so will take longer to rear and be less productive if captive bred. For all these reasons, we therefore predicted that species with small body sizes should be less expensive. We also expected that more colourful species would be more attractive as cage birds [40–42], and hence predicted that drabber species would sell for lower prices]. Finally, species are traded for singing competitions in Asia, and so we hypothesised that species with less attractive songs would fetch a lower price.

## Methods

### Data

To obtain data on the occurrence and price of bird species in Taiwanese pet shops, we (SS) conducted non-structured interviews between August and November 2012. Data on the locations of pet shops were obtained from the Commerce Industrial Services Portal, telephone directories, previous surveys and pet shop-related blogs. In this way, we located a total of 154 pet shops. This excludes game pigeon stores, pet product-only and aquarium shops. During the survey, we found that some of the shops had closed down, while some owners declined to respond to the survey. Therefore a total of 72 shops were surveyed in seven cities. Of the final survey sites, 32 were in Taipei City (and New Taipei City), seven in Hsinchu City, 11 in Taichung City, one in Taitung City, 14 in Kaohsiung City, five in Pingtung County and two shops in Hualien City. All shops were visited at least once with the aim of obtaining data on three variables: (i) the identity of species displayed for sale, (ii) their price (in Taiwanese Dollars, or TWD) and (iii) the numbers of birds of each species displayed. During each visit, SS first enquired about the identity of the species available for sale, their price and then counted and recorded the number of birds of each species displayed.

Ethics approval for the study was given by the ZSL Ethics Committee. The non-structured interviews of the shop owners did not involve questions about their personal information or identity. This is because the sources of birds in trade in Taiwan are frequently illegal, and therefore owners will not provide signed documents and/or written consent. We aimed to record information on the numbers of species, their identity and their price in the shops, and we obtained shop owners' verbal agreements to obtain such information. The consent procedure was documented in writing by SS and had been approved by the Ethics Committee.

We identified all taxa to species-level except for three white-eye species (Oriental White-eye *Zosterops palpebrosus*, Lowland White-eye *Z*. *meyeni* and Japanese White-eye *Z*. *japonicus*). The three White-eye species could be identified only by the owners, but the names they give can be unreliable as the sources of some of the White-eye species can be illegal. Two Myna species (Javan Myna *Acridotheres javanicus* and Great Myna *A*. *grandis*) were identified to species level, but these species are frequently stocked in mixed species cages with more than 50 birds in each cage. This makes it difficult to count the number of each species in a cage, and we therefore assumed that all mynas in any given cage belonged to the majority species. Sometimes the cages are packed and stacked on top of one-another, which makes species-level identification difficult. In this case, we assumed that all mynas in stacked cages belonged to the majority species from the visible cages. Where owners or staff were not prepared to reveal species identities, we identified birds to species level using monographs and bird guides, often from photographs taken by SS. In this way, a total of 247 bird species were identified. We used only species recorded in the first visit to each shop for analysis (n = 221) to standardise estimates of numbers for sale per shop. Furthermore, the only two Nearctic species recorded for sale (Northern Mockingbird *Mimus polyglottos* and Red-bellied Woodpecker *Melanerpes carolinus*) were removed to ease regional comparisons. One further species (Taiwan Hwamei *Garrulax taewanus*) was excluded because an estimate of price was not available. For PGLS analysis (see below) we excluded Red-collared Lorikeet *Trichoglossus rubritorquis* when phylogenetic trees were selected based on [43], because the authors treat it as a subspecies of Rainbow Lorikeet *Trichoglossus haematodus* (resulting in n = 217).

We based our analysis on the global taxonomic list of 9,993 extant bird species from Jetz *et al*. [43]. The scientific names of the species followed Birdlife Version 3 and IOC Version 2.7 taxonomies, family names followed the Birdlife guide and the order names followed Sibley & Monroe (1990) [43]. We identified species as native or alien in Taiwan based on the CWBF Checklist of the Birds of Taiwan [44], adjusted by excluding transient migrants, pelagic seabirds and vagrants.

We collated information for each of the species on the following variables:

1. Geographical range size (km^2^): a measure of native geographical range extent [45,46], acquired from the data applied in [47][47]. Maps of total native breeding area for all species were converted to equal area grid polygons with a cell size of 96.3 km x 96.3 km. This specified a scale identical to 1° grids at the equator. The breeding range of each species was estimated by summing the areas of the cells in which they occurred [47,48].
2. Region of origin: the biogeographic region from which the native populations of alien species originate, defined using the World Wildlife Fund eco-regions map [48,49]. Species were assigned to the region in which the largest part of their geographic range fell.
3. Body mass (g): a measure bird species body size from Dunning [50], augmented by data used in Olson *et al*. [51].
4. Conservation status of species: The conservation status of the identified species was obtained from the IUCN Red List of Threatened Species [52]. Species recorded in our survey were classified into the following categories, treated as a multi-level categorical variable: least concern (LC), near threatened (NT), vulnerable (VU) and endangered (EN). All categories were tested in our univariate analysis. We then separated species by whether they were in the threatened categories (NT, VU and EN) or not (LC) in our multivariate analysis. None of the species in our survey of pet shops was data deficient (DD), critically endangered (CR) or extinct in the wild (EW).
5. International trade status: The status of the identified species was obtained from Convention of International Trade in Endangered Species of Wild Fauna and Flora (CITES Appendices I, II and III). We separated species by whether they were included in Appendices I, II and III (scored 1) or not (scored 0).
6. Colour: Adult males of the nominal subspecies in breeding plumage (or females for species with reverse sexual dimorphism) were assessed for colouration on the basis of the plates in the Handbook of the Birds of the World. The colour of fourteen body parts of the bird (bill, face, cheek, head, throat, breast, belly, flank, back, wings, tail, rump, vent and legs) was assessed by eye, and the presence of eleven colours (blue, green, red, orange, brown, pink (including purple and violet), yellow, black, grey, white and ivory (pale flesh colour)) was scored. Two metrics were calculated from these data: 1) the percentage of the body surface covered by each colour, calculated over the fourteen body parts of the bird scored; and 2) colour diversity, calculated as the number of different colours on a given species (excluding colours covering <3% of the body).
7. Song attractiveness: We used the number of song tracks lodged for a given species on the Xeno-Canto website [www.xeno-canto.org] to derive a metric of the attractiveness of bird song to humans (following [53]). Xeno-Canto contains more than 115,000 independent recordings of bird songs, which cover about 80–85% of the extant species, with up to 335 recordings per species. Only two of the species in our data set do not have any song recordings on Xeno-Canto, while the range for the remainder is 1–162 records. We used the glm function in R v.3.0.3 [54] to model the log+1 number of songs lodged on Xeno-Canto as a function of log geographic range size. It has been shown that larger residuals are correlated with the characteristics of songs associated with attractiveness to people [53].

Of the nine predictor variables in the univariate analysis (see below), bird numbers, species status, region of origin, IUCN list and CITES listing, body mass and each colour, breeding range size and the attractiveness of bird song are available for 217 species. We log-transformed the data on body mass, number of birds of each species for sale, percentage of covered colour and their price, and applied arcsine square root transformations to the percentage of body surface covered in each colour.

### Analysis

All analyses were conducted in R version 3.0.3 [54].

We constructed phylogenetic generalized least squares (PGLS) models for the prices of species recorded in Taiwanese pet shops (response variable) compared with all nine univariate predictor variables, using the *pgls* function in the package *caper* (region of origin, species status, IUCN status, and CITES list were treated as categorical variables). The *pgls* model specifies the structure of the variance—covariance matrix reflecting the phylogenetic associations among the species, and also includes an estimated parameter (λ) that governs the strength of phylogenetic signal in the dependent variable. Thus, *pgls* does not require the *a priori* assignment of phylogenetic signal but instead allows the data to dictate its magnitude in the statistical model [55]. Clearly, price is not an evolved trait, but it is correlated with species traits that are (see Results), and our phylogenetic approach provides a way to analyse the influence of these traits under a specified evolutionary hypothesis (i.e. a phylogeny) in a way that accounts for the non-independence of species in terms of price (c.f. [56]). The significance of colour in univariate analysis was assessed after a Bonferroni correction, given that we test for the effects of 11 different colours. Collinearity amongst the different colour variables was low. Ivory colouration was found to have the strongest correlation with other colour variables; it exhibits the strongest positive correlation to brown (Pearson’s r = 0.37, n = 217, P < 0.001) and negative to black (Pearson’s r = −0.33, n = 217, P < 0.001). To find the most likely model for bird price, we fitted a global PGLS model including all predictors. We then used the *dredge* and *model*.*avg* functions (package *MuMIn*) to fit all possible models from these predictor variables, to identify the most likely models, and to calculate Akaike weights and variable importance (the sum of the Akaike weights across all models including that variable) based on the Akaike Information Criterion corrected for small sample sizes (AICc).

We based our phylogenetic analyses on the phylogenetic tree for birds proposed by [43]. The structure of this phylogenetic tree is not known for certain, and so we incorporated uncertainty over the true phylogenetic relationship by repeating our analyses over a number of different phylogenetic trees for our species, sampled at random from www.birdtree.org [43]. Initially, we used a sample of 100 random trees to calculate our univariate relationships. We then used ten trees (the smaller sample due to constraints on computer time necessary for the multi-model approach over hundreds of possible models) to identify the most likely set of models (those with ΔAICc < 4 of the best model) from our global PGLS model, and a further random selection of 100 phylogenetic trees to assess the fit and importance of models in this set. Thus, the simulation produced median estimates (over these trees) of the parameters of the best-fit models incorporating phylogenetic uncertainty. This approach allowed us to provide estimates of the variance in regression coefficients, which we calculated as the 5^th^ and 95^th^ percentiles in the coefficients across the 100 runs.

## Results

The total number of birds for sale in the pet shops in our survey across all 217 species was 26,165, of which 7,612 individuals were of species alien to Taiwan (n = 159). The maximum recorded number of birds per species was 7,420 for Red Turtle Dove *Streptopelia tranquebarica*, while the highest single bird count in a single shop was 4,000 individuals, for this same species. The ten most abundant species in Taiwanese pet shops are listed in Table 1. These ten species comprised 80% of all individuals for sale, while the five native species in Table 1 account for 70% of all birds for sale. More than half of the alien bird individuals for sale came from just five species (Table 1). 67 of the alien species (2,778 individuals) for sale in the pet shops were parrots.

**Table 1:** The ten traded bird species with the largest numbers and ten with highest price recorded for sale in Taiwanese pet shops in our survey.

Family	*Species*	Number of birds	Price (US$)	Status
Columbidae	*Streptopelia tranquebarica*	7420	1.63	Native
Zosteropidae	*Zosterops japonicus*	6381	35.50	Native
Estrildidae	*Lonchura punctulata*	2649	1.50	Native
Psittacidae	*Melopsittacus undulatus*	956	7.70	Alien
Estrildidae	*Padda oryzivora*	928	10.47	Alien
Sturnidae	*Acridotheres grandis*	722	10.00	Alien
Sturnidae	*Acridotheres fuscus*	492	6.83	Alien
Pycnonotidae	*Pycnonotus sinensis*	465	10.03	Native
Estrildidae	*Lonchura striata*	437	2.40	Native
Estrildidae	*Erythrura gouldiae*	405	13.03	Alien
Psittacidae	*Guaruba guarouba*	5	8000.00	Alien
Psittacidae	*Cacatua leadbeateri*	2	7500.00	Alien
Psittacidae	*Amazona oratrix*	1	6666.67	Alien
Psittacidae	*Ara macao*	1	4000.00	Alien
Psittacidae	*Cacatua moluccensis*	2	3333.33	Alien
Psittacidae	*Ara chloropterus*	2	2083.33	Alien
Psittacidae	*Cacatua ophthalmica*	1	2000.00	Alien
Psittacidae	*Primolius auricollis*	5	1916.67	Alien
Psittacidae	*Ara ararauna*	18	1818.17	Alien
Psittacidae	*Pionites leucogaster*	4	1777.77	Alien

Table with numbers of birds recorded for sale, their price (US$) and whether species are alien or native to Taiwan (Status).

The prices of the 217 identified species sold varied widely, from US$0.86 (at an exchange rate of US$1:TWD30) per individual for Eurasian Tree Sparrow *Passer montanus* to US$8,000 for a single Golden Parakeet *Guaruba guarouba*. The total market value of displayed birds in the surveyed pet shops during the survey time was more than US$0.8 million, of which more than US$0.5 million (> 65%) was contributed by alien species.

**Fig 1:**
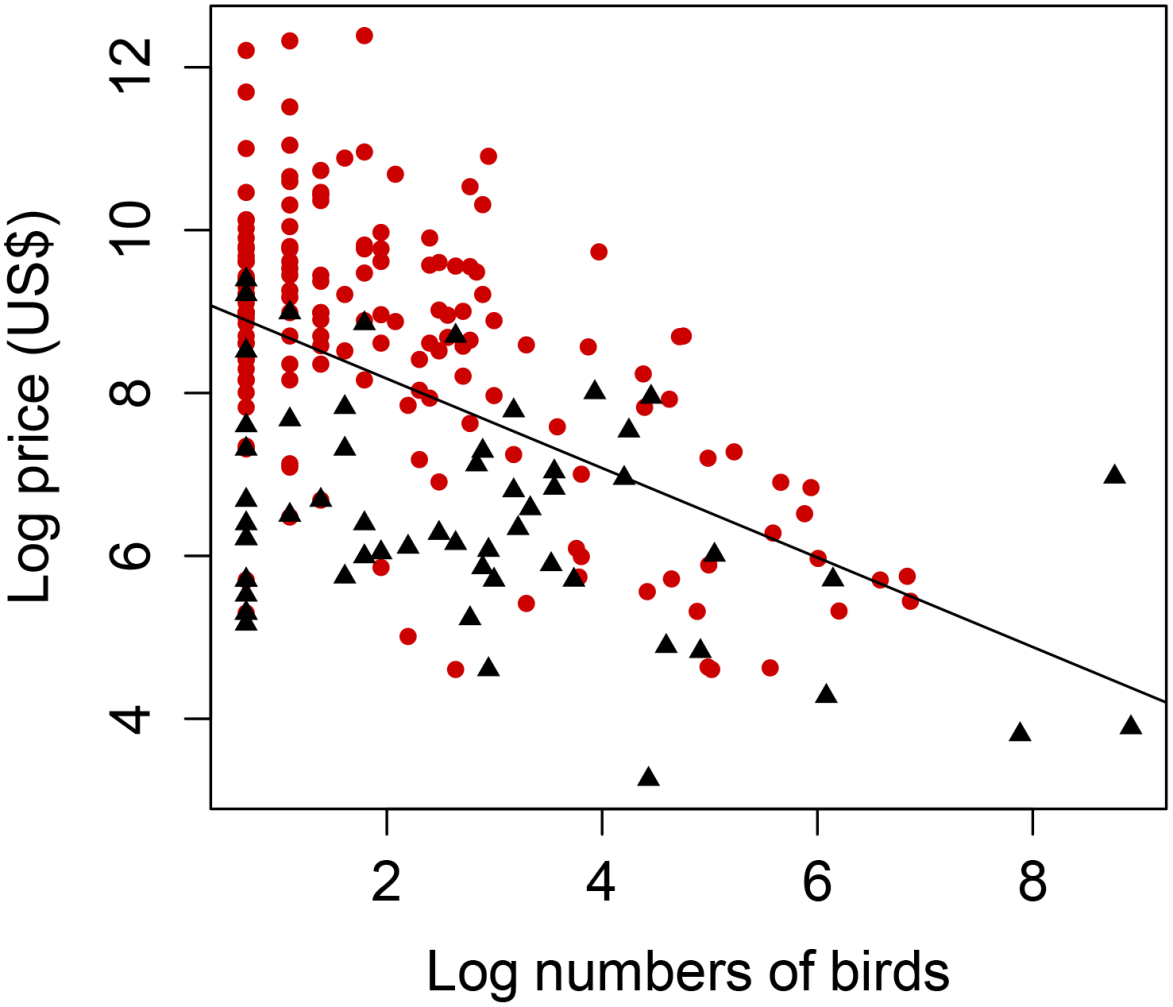
The relationship between log price and log abundance of alien and native species recorded in Taiwan. Red circles indicate the alien species and black triangles indicate the native species for sale in Taiwanese pet shops. The line indicates the best fit relationship between log price (in Taiwanese Dollars, or TWD) and log numbers irrespective of native or alien status (r^2^ = 0.27, n = 217). Both transformations are natural logarithms.

Univariate PGLS models revealed that the prices of birds for sale in pet shops were related to the number of birds of each species for sale (Fig 1), body mass, whether or not the species is alien to Taiwan (Fig 1), whether or not the species is listed in CITES Appendices, breeding geographic range size, song attractiveness (Table 2) and colour (the percentage of a species’ body that was yellow or grey; Table 3). There was also an effect of a species’ categorisation on the IUCN Red List (adjusted r^2^ ± s.e. = 0.084 ± 0.0008, F_3,177_ ± s.e. = 5.41 ± 0.059, p ± s.e. = 0.002±0.0001, λ ± s.e. = 0.952±0.0007), and a marginal effect of biogeographic realm of origin (adjusted r^2^ ± s.e. = 0.091 ± 0.0007, F_4,88_ ± s.e. = 2.21 ± 0.01, p ± s.e. = 0.076 ± 0.002, λ ± s.e. = 0.95 ± 0.0004): in general, less threatened species and species from realms closer to Taiwan (Palaearctic, which include Taiwanese native species) were cheaper.

**Table 2:** The relationship between the log-transformed prices of traded bird species in Taiwanese pet shops and the predictor variables.

Variables	Estimate	t value	Lambda (λ)	p value
**Log number for sale**	−0.14 [−0.15, −0.14]	−6.91 [−7.01, −6.77]	0.94 [0.94, 0.95]	< 0.001
**Log mass**	0.93 [0.89, 0.98]	6.27 [5.83, 6.89]	0.93 [0.92, 0.94]	< 0.001
**Log geographic range**	−0.18 [−0.2, −0.16]	−2.67 [−2.95, −2.44]	0.96 [0.95, 0.97]	0.008 [0.004, 0.01]
**Song attractiveness**	−0.09 [−0.1, −0.08]	−2.09 [−2.32, −1.91]	0.97 [0.96, 0.97]	0.03 [0.02, 0.05]
**Status (native/alien)**	−0.54 [−0.55, −0.52]	−2.5 [−2.7, −2.28]	0.95 [0.95, 0.96]	0.01 [0.007, 0.02]
**CITES (listed/not)**	0.69 [0.66, 0.72]	3.92 [3.71, 4.09]	0.94 [0.93, 0.95]	<0.001
**Realm (Australasia, n = 38)**	0.06 [0.04, 0.1]	0.27 [0.16, 0.41]	0.95 [0.95, 0.96]	0.78 [0.67, 0.86]
**Realm (Indo-Malay, n = 64)**	−0.35 [−0.39, −0.31]	−1.42 [−1.57, −1.23]	0.95 [0.95, 0.96]	0.15 [0.11, 0.21]
**Realm (Neotropic, n = 41)**	0.4 [0.37, 0.43]	1.2 [1.09, 1.29]	0.95 [0.95, 0.96]	0.23 [0.2, 0.27]
**Realm (Palearctic, n = 52)**	−0.45 [−0.47, −0.42]	−2.16 [−2.34, −1.95]	0.95 [0.95, 0.96]	0.03 [0.02, 0.05]

The averaged coefficients (median estimates [5^th^, 95^th^ percentiles]) from univariate analysis on the relationship between the log-transformed price of bird species for sale in Taiwanese pet shops (n = 217) and the predictor variables in the first column, were calculated over 100 randomly selected likely phylogenetic trees (see Methods for more details). Parameter Lambda (λ) indicates the strength of phylogenetic signal in the dependent variable (price of birds for sale in Taiwan).

**Table 3:** The relationship between the price of traded bird species in Taiwanese pet market and the percentage of a given colour found on the species, and colour diversity.

Colour (percentage of colour)	Estimate	t value	p value	Lambda (λ)
**Black**	3.95 [3.51, 4.34]	2.03 [1.79, 2.27]	0.04 [0.02, 0.07]	0.94 [0.93, 0.95]
**Blue**	1.45 [1.14, 1.74]	0.93 [0.76, 1.11]	0.34 [0.26, 0.44]	0.94 [0.93, 0.95]
**Green**	−2.12 [−2.64, −1.53]	−1.61 [−2.06, −1.14]	0.1 [0.04, 0.25]	0.94 [0.94, 0.95]
**Red**	4.49 [4.15, 4.82]	2.41 [2.24, 2.61]	0.01 [0.009, 0.02]	0.94 [0.93, 0.95]
**Orange**	−2.3 [−2.54, −1.86]	−1.31 [−1.44, −1.04]	0.19 [0.15, 0.29]	0.94 [0.94, 0.95]
**Yellow**	4 [3.72, 4.28]	2.89 [2.69, 3.06]	0.004* [0.002, 0.007]	0.95 [0.94, 0.95]
**Brown**	−2.69 [−3.05, −2.24]	−1.75 [−2.04, −1.46]	0.08 [0.04, 0.14]	0.94 [0.93, 0.95]
**Pink**	5.25 [4.55, 6.25]	2 [1.66, 2.57]	0.04 [0.01, 0.09]	0.95 [0.94, 0.95]
**White**	−0.55 [−1.17, 0.16]	−0.22 [−0.47, 0.06]	0.82 [0.63, 0.98]	0.94 [0.93, 0.95]
**Grey**	−6.2 [−6.64, −5.79]	−3.32 [−3.55, −3.12]	0.001*[0.0004, 0.002]	0.94 [0.93, 0.95]
**Ivory**	0.14 [−0.44, 0.82]	0.05 [−0.17, 0.33]	0.91 [0.72, 0.98]	0.94 [0.93, 0.95]
**Colour Diversity**	0.007 [0.002, 0.01]	0.26 [0.07, 0.59]	0.78 [0.55, 0.94]	0.94 [0.04, 0.95]

The averaged coefficients (median estimates, [5^th^, 95^th^ percentiles]) from univariate analysis on the relationship between the log-transformed price of bird species for sale in Taiwanese pet shops and the arcsine square root transformed percentage of a given colour found on the species, and colour diversity (in the first column), were calculated over 100 likely phylogenetic trees. Parameter Lambda (λ) indicates the strength of phylogenetic signal in the price of birds for sale in Taiwan.

*significant applying a Bonferroni correction for multiple tests; N = 217 in each case.

Model selection on the global model for the price of birds in Taiwanese pet shops identified 13 models for which the median ΔAICc across the ten trees was <4 relative to the most likely model (Table 4). As an indicator of fit, the variables in the most likely model explained 61% of the variance in bird price. The simulation over 100 randomly chosen but likely phylogenetic trees for each of these best 13 PGLS models recovered four variables—body mass, number of birds for sale, whether or not species are alien to Taiwan and song attractiveness—as present in all of the possible model combinations. The presence of yellow coloration also had relatively high variable importance (median importance [5^th^, 95^th^ percentiles] = 0.96 [0.93, 0.98]). The variable importance of the CITES trade status is less than the previous five predictors but higher than the other variables (median importance [5^th^, 95^th^ percentiles] = 0.71 [0.65, 0.79]). Thus, cheaper birds in Taiwanese pet shops tend to be physically small-bodied, not yellow in colour and not attractive singers, as well as highly available in the local markets, Taiwanese natives, and not included in any CITES Appendices.

**Table 4:** Model coefficients in PGLS models for determinants of the price of birds in Taiwanese pet shops.

**Intercept**	**Whether listed in CITES appendices**	**Grey colour**	**Log breeding range**	**Log numbers**	**Log mass**	**IUCN status**	**Song attractiveness**
2.09 [1.99, 2.2]	0.28 [0.26, 0.31]	−2.61 [−2.9, −2.29]		−0.1 [−1.1, −1.1]	0.61 [0.57, 0.66]		−0.07 [−0.08, −0.06]
1.99 [1.89, 2.1]	0.28 [0.25, 0.31]			−0.1 [−0.1, −0.1]	0.64 [0.59, 0.69]		−0.07 [−0.07, −0.06]
2.1 [2, 2.21]	0.2 [0.251, 0.3]	−2.57 [−2.82, −2.24]		−0.1 [−1.1, −1.1]	0.61 [0.56, 0.66]	0.06 [0.04, 0.08]	−0.07 [−0.08, −0.06]
2.23 [2.1, 2.36]	0.28 [2.56, 0.31]	−2.61 [−2.89, −2.28]	−0.02 [−0.2, −0.008]	−0.1 [−1.1, −1.1]	0.61 [0.56, 0.66]		−0.07 [−0.08, −0.06]
2.02 [1.93, 2.14]		−2.51 [−2.83, −2.19]		−0.11 [−0.11, −0.1]	0.67 [0.62, 0.71]		−0.07 [−0.07, −0.06]
1.99 [1.9, 2.11]	0.27 [0.23, 0.3]			−0.1 [−0.11, −0.1]	0.64 [0.59, 0.69]	0.07 [0.04, 0.08]	−0.06 [−0.07, −0.05]
1.92 [1.82, 2.04]				−0.11 [−0.11, −0.11]	0.69 [0.65, 0.74]		−0.06 [−0.07, −0.05]
2.13 [2.01, 2.25]	0.27 [0.24, 0.3]		−0.02 [−0.03, −0.009]	−0.1 [−0.11, −0.1]	0.64 [0.59, 0.69]		−0.07 [−0.07, −0.06]
2.04 [1.94, 2.15]		−2.46 [−2.77, −2.12]		−0.11 [−0.11, −0.1]	0.66 [0.61, 0.7]	0.07 [0.05, 0.09]	−0.06 [−0.07, −0.05]
1.94 [1.83, 2.06]				−0.11 [−0.11, −0.11]	0.68 [0.63, 0.73]	0.08 [0.05, 0.09]	−0.06 [−0.07, −0.05]
2.16 [2.05, 2.27]	0.29 [0.26, 0.31]	−3.46 [−3.73, −3.14]		−0.1 [−0.1, −0.1]	0.61 [0.56, 0.65]		−0.07 [−0.08, −0.07]
2.15 [2.02, 2.29]	0.28 [0.24, 0.3]	−2.58 [−2.85, −2.24]	−0.009 [−0.01, 0.003]	−0.1 [−0.1, −0.1]	0.61 [0.56, 0.66]	0.05 [0.03, 0.07]	−0.07 [−0.08, −0.06]
2.19 [2.06, 2.32]		−2.51 [−2.82, −2.18]	−0.02 [−0.03, −0.01]	−0.11 [−0.11, −0.1]	0.66 [0.61, 0.7]		−0.07 [−0.07, −0.06]
**Importance**	0.71 [0.65, 0.79]	0.6 [0.5, 0.7]	0.23 [0.16, 0.29]	1	1	0.29 [0.22, 0.35]	1
**Status (native/exotic)**	**Yellow colour**		**Model ranks**		**ΔAIC**	**Lambda** (λ)	**Akaike weight**
		**Rank 1**	**Rank 2**	**Rank 3**			
−0.24 [−0.27, −0.22]	2.74[2.56, 2.95]	97	3	0	0 [0, 1]	0.92 [0.9, 0.93]	0.18 [0.154, 0.21]
−0.25 [−0.27, −0.22]	3.17 [0.002, 3.36]	3	84	9	0.71 [0.02, 1.44]	0.92 [0.9, 0.93]	0.12 [0.08, 0.17]
−0.24 [−0.27, −0.21]	2.66 [2.48, 2.85]	0	11	71	1.37 [0.98, 1.88]	0.92 [0.91, 0.94]	0.09 [0.07, 0.11]
−0.24 [−0.27, −0.22]	2.7 [2.52, 2.91]	0	0	2	1.82 [1.43, 2.11]	0.92 [0.9, 0.94]	0.07 [0.06, 0.09]
−0.24 [−0.27, −0.21]	2.74 [2.54, 2.95]	0	2	9	1.86 [1.33, 2.48]	0.92 [0.91, 0.94]	0.07 [0.04, 0.09]
−0.25 [−0.27, −0.23]	3.08 [2.91, 3.24]	0	0	8	2.03 [1.31, 2.66]	0.92 [0.91, 0.93]	0.06 [0.04, 0.08]
−0.25 [−0.27, −0.22]	3.16 [2.98, 3.36]	0	0	1	2.41 [1.49, 3.23]	0.92 [0.91, 0.94]	0.05 [0.03, 0.07]
−0.25 [−0.27, −0.22]	3.13 [2.96, 3.32]	0	0	0	2.54 [1.83, 3.10]	0.92 [0.91, 0.93]	0.05 [0.03, 0.06]
−0.24 [−0.27, −0.21]	2.65 [2.45, 2.85]	0	0	0	2.96 [2.13, 3.92]	0.93 [0.91, 0.94]	0.04 [0.02, 0.05]
−0.24 [−0.27, −0.21]	3.04 [2.87, 3.24]	0	0	0	3.47 [2.43, 4.31]	0.93 [0.91, 0.94]	0.03 [0.02, 0.04]
−0.28 [−0.31, −0.26]		0	0	0	3.48 [2.69, 4.28]	0.91 [0.89, 0.93]	0.03 [0.02, 0.04]
−0.24 [−0.27, −0.21]	2.65 [2.47, 2.85]	0	0	0	3.51 [3.03, 4.08]	0.92 [0.91, 0.93]	0.03 [0.02, 0.04]
−0.25 [−0.27, −0.21]	2.7 [2.49, 2.91]	0	0	0	3.53 [2.84, 4.42]	0.92 [0.91, 0.94]	0.03 [0.02, 0.04]
1	0.96 [0.93, 0.98]						

Model coefficients (median estimates, [5^th^, 95^th^ percentiles]) in PGLS models (ΔAIC from the best model is less than 4) for determinants of the price of birds in Taiwanese pet shops, were calculated over 100 randomly selected likely phylogenetic trees. Model ranks (top 3 rankings shown) are based on ΔAIC. Parameter Lambda (λ) indicates the strength of phylogenetic signal in the price of birds for sale in Taiwan for each likely model. IUCN status divided species by whether they are classed in the category of least concern.

The 13 most likely models also included effects of IUCN listing, breeding area and grey colour, although these variables had lower importance than the others in the model (Table 4). Prices tended to be lower for species less threatened on IUCN status, with larger geographic range sizes and more grey colouration.

**Fig 2:**
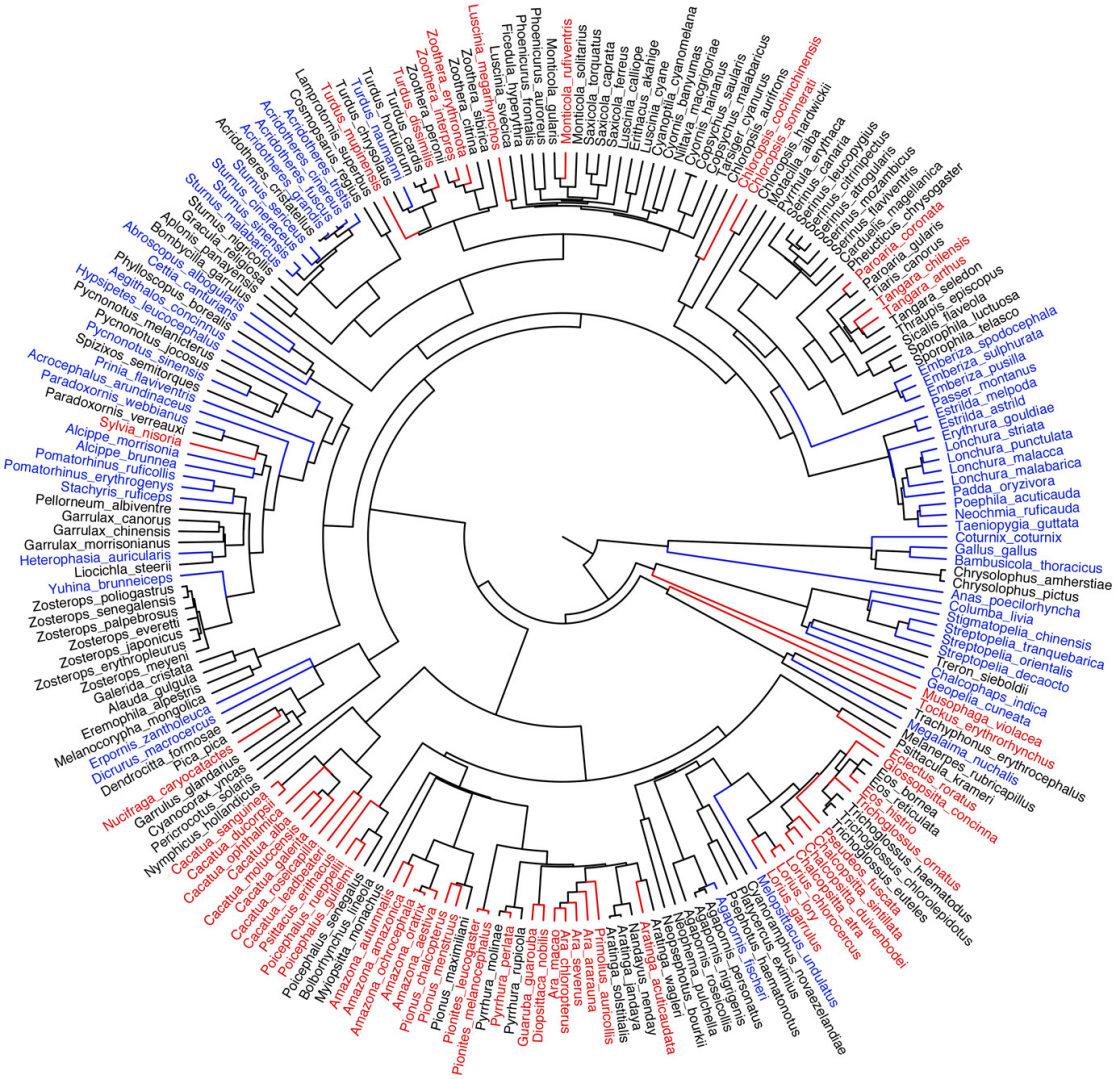
A phylogeny of the bird species included in our analysis. Species were classed in three ranges by the price for every identified species (n = 217). Expensive bird species (species in the top quartile for price; n = 56) are marked in red; cheap bird species (species in the bottom quartile for price; n = 55) are marked in blue. The phylogeny was generated from birdtree.org (Jetz et al. 2012), visualised in FigTree v1.4.0.

The analyses of univariate models (Tables 2 and 3), the global PGLS model (median λ [5^th^, 95^th^ percentiles] = 0.92 [0.90, 0.94] and the 13 most likely PGLS models (Table 4) all identified a high degree of phylogenetic correlation (Pagel’s λ) in the models: price tended to be similar for closely related species (Fig 2).

## Discussion

The most important correlate of variation in the number of invasive alien species across countries is currently the volume of imported goods [17], suggesting that international trade is now the primary driver of vertebrate species introductions. The bird trade in East and South-East Asia is economically lucrative and active. The invasion pathway for birds has been well-studied in Western countries, but in Eastern countries birds are traded for different reasons, and trade concerns different species with different characteristics traded in different quantities [20,57,58]. It is particularly important to understand the trade in response to Eastern market demands, in comparison to Western trade, which has been curtailed since the unpopularity of past Acclimatisation Society activity and, currently, by wildlife trade bans. This is the primary motivation behind our analyses of the variation in price of bird species for sale in Taiwanese pet shops. The price of birds is a good indicator of the constraints and demands of the pet market, which allows us to identify the characteristics of traded species, and hence a key element in the process of human-mediated bird invasions in East and South-East Asia.

Our analyses revealed that the price of species in pet shops is strongly associated with their availability. Thus, species that are for sale in large numbers, are native to Taiwan, and with healthy wild populations available for international trade (as assessed by CITES) all fetch a relatively low price in Taiwan. The first two of these variables were present in all of the identified most likely models, while the third was present in most of these models (Table 4). All also have model-averaged regression coefficients that differ significantly from zero with a high degree of confidence (Tables 2 and 4). For example, the Eurasian Tree Sparrow *P*. *montanus*, that is a common resident native species in Taiwan [59], is not included in CITES Appendices, and is commonly sold species in Taiwanese pet shops, it costs around US$0.86 per individual. In contrast, the Golden Parakeet *G*. *guarouba* is native to South America (i.e. alien), is included on CITES Appendix l (at the time of our survey), and costs around US$8,000 per individual.

Alien species are probably more expensive because of reduced availability, as there are fewer alien birds for sale in Taiwanese pet shops than natives. Alien species must be imported from overseas [60], and higher prices presumably reflect importation costs (Fig 1). Moreover, higher prices could also reflect the scarcity of captive-bred individuals, particularly for species included on CITES Appendix l. The price inflation for species as alien versus as native is also illustrated by the comparative prices of species in bird markets in different countries. For example, the Red-crested Cardinal *Paroaria coronata* and Saffron Finch *Sicalis flaveola* are both native to South America. Their prices in local markets in Brazil were US$83.33 and US$16.11, respectively (where the lowest recorded price of any species is US$3.33) [61], in comparison to US$750 and US$122.23 for the same species in Taiwanese pet markets (where the lowest recorded price of any species is US$0.86).

Whether or not a species was listed in the CITES appendices (and therefore restricted in international trade) has significant effects on the price of species in the Taiwanese pet market (Tables 2 and 4), albeit that the variable importance for CITES listing is less than that of the five primary predictors of price (Table 4). The sources of species in pet shops (captive-bred or wild-caught) are often unclear [28], but the bulk of pet trade is made up of wild-caught species [16,27,57]. Not only is the international trade of CITES-listed species restricted, but they are also likely to fall under local regulations such as the Wildlife Conservation Act in Taiwan [20,62], and so we would expect an effect of CITES listing on price. Courchamp *et al*. [32] also showed that CITES listed amphibian and reptile species fetched a higher price in French trade than equivalent non-CITES species.

Larger-bodied species sell for a higher price in the Taiwanese pet market (Tables 2 and 4). Large-bodied species tend to have lower reproductive rates, require more food and space, and live at lower densities in the wild than smaller-bodied species [63]. All of these factors will increase the costs of dealing in these species for pet shop owners, bird breeding farms, and bird catchers and export/importers. Large-bodied species also tend to be long-lived. For example, some of the larger parrot species have lifespans similar to humans [64–66]. Buying such a bird may be a once in a lifetime purchase, raising the price that is likely to be charged. A well-trained parrot may command a very high price, especially if it can imitate human language [42]. This probably explains why species in birds such as macaws (*Ara*), amazons (*Amazona*) and cockatoos (*Cacatua*) fetch a high price in Taiwan (Fig 2).

We predicted a positive effect of the attractiveness of a bird’s song on its price, because many species are traded for their ability as songsters and previous analysis showed that species for sale in Taiwanese pet shops had more aesthetically-pleasing songs than expected by chance [67]. In fact, we found the reverse relationship. It is possible that a higher demand for species with attractive songs has led to an abundance of such species in the market, and hence an associated lower price. However, the relationship is more likely to be a consequence of some birds, such as parrots, not being traded for their song, but for other reasons, such as the ability to mimic human voice [42]. If we limit analysis to song birds (species in the order Passeriformes), then the negative relationship between song attractiveness and price disappears (univariate PGLS: estimate median [5^th^, 95^th^ percentiles] = –0.03 [–0.04, –0.01], t = –0.48 [–0.66, 0.22], p = 0.063 [0.51, 0.82], λ = 0.90 [0.88, 0.93).

We also found some evidence that the price of bird species in Taiwan is influenced by plumage colour, and specifically that species with a higher proportion of yellow colouration fetch a higher price, while species with a higher proportion of grey colouration fetch a lower price (Tables 3 and 4). Cultural factors may be key in driving such colour preferences [68]. For example, the colour grey has associations with low value in Asian countries (e.g., China and Japan), but associations with high quality and expense in the U.S. [69]. In traditional Chinese culture, nature is composed of five elements—wood, fire, earth, metal and water—and each element has a colour and a compass point associated with it [70]. Yellow exemplifies the earth and represents the centre of the compass, and is therefore of high importance. Yellow was the colour for Imperial China: yellow was an exclusive colour of the imperial family during some Dynasties (e.g., the Qing dynasty)[71] and is a colour venerated in Buddhism. Taiwan has a Chinese-influenced culture, and it seems that yellow plumage convinces people to pay a higher price for bird species in the Taiwanese pet trade.

The price of birds was apparently less affected by variables relating to the realm of origin or native breeding range size. The effects of realm of origin and native breeding range size, which we predicted would affect price through availability, are presumably captured more directly by other availability measures, such as number for sale and whether or not a species is native.

Phylogenetic analysis identified a strong phylogenetic correlation in bird price. Related species thus tend to vary in price in a similar manner, which suggests that the factors that determine price are similar for related species. In fact, at least three of the four main predictors of price would be expected to show strong phylogenetic autocorrelation: body mass [72], threat status [73], and alien status, the last given that several of the families with birds for sale in Taiwan have no native species (e.g. parrots, turacos, tanagers). The phylogenetic correlation for analysis of the fourth main predictor—numbers of birds for sale—is also strong (Table 2). However, why this should be so is less clear, especially given that the abundances of bird species in the wild tend to show relatively low phylogenetic correlation (e.g. [74,75]). It may be that some bird taxa are easier to catch, survive better in transportation, and/or are easier to breed in captivity.

### Implications

Alien invasive species have a wide range of environmental impacts [76], and are one of the largest threats to the persistence of native species [8]. In Taiwan, for example, the endemic Styan’s bulbul *Pycnonotus taivanus* is categorised as vulnerable on the IUCN Red List due to hybridization with the introduced alien Chinese bulbul *P*. *sinensis*. The aim of this study was to provide insight into the contribution of the cage bird trade to bird invasions. Assuming that cheaper birds are more likely to be liberated (or conversely that more expensive birds are less likely to be so), and species more commonly sold also more likely to escape or be released, then one might conclude from our results that pet shop birds represent a low risk for alien bird invasions in Taiwan. The cheapest species in pet shops are native, as are the species most likely to be bought for prayer release. Prayer birds typically need to be readily and cheaply available [77]. Prayer animals are released regardless of whether or not they are alien to Taiwan, but alien bird species are often priced higher in the shops. Thus, cheaper native species are presumably much easier to source for shops, especially for sale for prayer release. The three most abundant species recorded in pet shops in our survey (Table 1) are all frequently released in religious ceremonies in Taiwan, and are native. All of this suggests that most of the individuals and species likely to be liberated in Taiwan do not present a risk of alien invasion because they are not alien.

Pet shop birds nevertheless still present a non-trivial invasion risk. Most of the species sold are alien (68%), and some of them are available for sale in reasonable numbers (Table 1) and at relatively low price (Figs 1 and 2). There is therefore the potential and opportunity for some alien species to be introduced to the wild, and in the sort of numbers required to promote population establishment (which is a positive function of the number of birds introduced [11,24,25,78]. This likelihood may be further increased by the dynamics of the bird trade. Robinson [79] showed that a high price for rare species can drive oversupply when traders and breeders rush to fulfil demand, with the result that market prices can decline rapidly. When demand is saturated and the market is low on profit, owners of breeding farms or pet shops would be more likely to release their birds (although not while species still sell for higher prices than other similar species). Alternatively, to minimise losses arising from the maintenance of unsold and unsellable birds in shops, pet shops may sell birds at a lower price for religious events.

Previous studies have also shown that when the sale of wild birds is prohibited (i.e. due to avian influenza), vendors were more likely to move to the illegal trade, or to release birds directly into the wild [80]. Many Asian countries have gone through outbreaks of avian flu since 2004 [81], and the bird trade in Taiwan is at risk of being affected by the disease or trade bans of nearby countries. A trade ban could therefore cause species to be released by vendors, whether alien or native. We suspect that species released this way would be likely to be those with lower economic value and to be more abundant in shops. However, maintaining higher value alien species costs more, and so owners may release the birds when their economic situation worsens. Therefore, we would not exclude the possibilities of pricier alien species also being released into the wild in these particular circumstances. We will explore the influence of price on the likelihood of introduction and establishment in a subsequent paper.

Solutions for preventing alien bird releases could, and do, target both the sources of the birds in trade, and the customers sustaining the trade. The numbers of traded individuals could be reduced through control of the sources of birds (wild capture and illegal imports); some such controls are already in place in Taiwan [20,62]. Educating the general population about the risk of releasing alien pet birds may have limited impact in the Asian context, because many purchases are for religious and cultural reasons and not only for companionship or status. Campaigns are already undertaken to discourage prayer release in Taiwan [77,82], although this practice continues to be commonplace there. An alternative approach (inspired by Venerable Bengkong Shi [83]) would be to co-opt the religious reasons for buying and releasing animals, and so gradually to replace the ceremonial release of randomly chosen (and potentially harmful) prayer animals with planned releases of native species for conservation purposes, such as re-introductions. This process could incorporate ritual ceremonies to pray for the welfare of the animals before they are released.

## Supporting Information

S1 Appendix The species recorded for sale in Taiwanese pet shops (first visit) in our survey, together with the variables used in our analysis. (CSV)

S2 Appendix Data on bird coloration used in our analysis. (CSV)
